# Decoding Gene Networks Modules That Explain the Recovery of *Hymenoglossum cruentum* Cav. After Extreme Desiccation

**DOI:** 10.3389/fpls.2020.00574

**Published:** 2020-05-15

**Authors:** Enrique Ostria-Gallardo, Giovanni Larama, Graciela Berríos, Ana Fallard, Ana Gutiérrez-Moraga, Ingo Ensminger, Patricio Manque, Luisa Bascuñán-Godoy, León A. Bravo

**Affiliations:** ^1^Laboratorio de Fisiología Vegetal, Centro de Estudios Avanzados en Zonas Áridas (CEAZA), La Serena, Chile; ^2^Scientific and Technological Bioresource Nucleus, Universidad de La Frontera, Temuco, Chile; ^3^Centro de Excelencia de Modelación y Computación Científica, Facultad de Ingeniería y Ciencias, Universidad de La Frontera, Temuco, Chile; ^4^Laboratorio de Fisiología y Biología Molecular Vegetal, Departamento de Cs. Agronómicas y Recursos Naturales, Facultad de Ciencias Agropecuarias y Forestales, Instituto de Agroindustria, Universidad de La Frontera, Temuco, Chile; ^5^Instituto de Ciencias Biomédicas, Universidad Autónoma de Chile, Santiago, Chile; ^6^Department of Biology, University of Toronto, Toronto, ON, Canada; ^7^Center for Integrative Biology, Universidad Mayor, Santiago, Chile; ^8^Laboratorio de Fisiología Vegetal, Universidad de Concepción, Concepción, Chile

**Keywords:** desiccation tolerance, gene discovery, homoiochlorophyllous, Hymenophyllaceae, neural network, temperate rainforest

## Abstract

*Hymenoglossum cruentum* (Hymenophyllaceae) is a poikilohydric, homoiochlorophyllous desiccation-tolerant (DT) epiphyte fern. It can undergo fast and frequent dehydration-rehydration cycles. This fern is highly abundant at high-humidity/low-light microenvironments within the canopy, although rapid changes in humidity and light intensity are frequent. The objective of this research is to identify genes associated to desiccation-rehydration cycle in the transcriptome of *H. cruentum* to better understand the genetic dynamics behind its desiccation tolerance mechanism. *H. cruentum* plants were subjected to a 7 days long desiccation-rehydration process and then used to identify key expressed genes associated to its capacity to dehydrate and rehydrate. The relative water content (RWC) and maximum quantum efficiency (*F*_v_/*F*_m_) of *H. cruentum* fronds decayed to 6% and 0.04, respectively, at the end of the desiccation stage. After re-watering, the fern showed a rapid recovery of RWC and *F*_v_/*F*_m_ (ca. 73% and 0.8, respectively). Based on clustering and network analysis, our results reveal key genes, such as *UBA/TS-N*, *DYNLL*, and *LHC*, orchestrating intracellular motility and photosynthetic metabolism; strong balance between avoiding cell death and defense (*CAT3*, *AP2/ERF*) when dehydrated, and detoxifying pathways and stabilization of photosystems (*GST*, *CAB2*, and *ELIP9*) during rehydration. Here we provide novel insights into the genetic dynamics behind the desiccation tolerance mechanism of *H. cruentum*.

## Introduction

A major problem that plants have faced since they colonized earth’s surface is the exposure to a high atmospheric demand of water. All current land plants have evolved structures, mechanisms and strategies to deal with dehydration. Among the different degrees of water deficit, desiccation is the most extreme form of dehydration. It occurs when most of the protoplasmic water is lost and only a very small amount of tightly bound water remains in the cell matrix (Djilianov et al., 2013). Despite the morphological innovations and physiological mechanisms to sustain their water balance (Bateman et al., 1998) most plants cannot live with less than ∼60–30% of water content (Dinakar and Bartels, 2013). However, an exceptional and small group of plants, called “resurrection plants,” can tolerate extreme desiccation (less than ∼5% of water content), and restore their metabolism completely when rehydrated (Ingram and Bartels, 1996; Alpert, 2000). Desiccation tolerant plants occur in phylogenetic distinct clades, along a wide range of environments. Thus, the acquisition of desiccation tolerance must have occurred multiple times and under a variety of environmental conditions (Farrant and Moore, 2011). Desiccation tolerance (DT) is a complex trait. Must integrate the perception and signaling of water loss, the protection of cellular components, and an efficient cellular repair activity (Alpert, 2000; Oliver et al., 2000).

During the last decades there has been a significant increase in our understanding of the desiccation tolerance of plants (Bartels et al., 1992; Bartels and Salamini, 2001; Moore and Farrant, 2015; Giarola et al., 2016; Lin et al., 2019). The physiological and metabolic component of DT combines processes observed in plants during drought stress and also seed maturation and dormancy (Farrant and Moore, 2011; Gechev et al., 2012). Evidence from biochemical to high throughput “omic” studies has indicated that changes in the expression and accumulation of various osmolites, late embryogenesis-abundant proteins (LEA), regulation of protein ubiquitination, and the efficient detoxification and antioxidant defense systems are key components for desiccation tolerance (Ramanjulu and Bartels, 2002; Dinakar and Bartels, 2013; Lin et al., 2019). Chaperones and other molecular shields (e.g., peroxiredoxins; heat shock proteins, HSPs; desiccation stress proteins, DSPs; early induced light protein like, ELIPs) are involved in the protection and stability of the physical properties of membrane and protein complexes (Chen et al., 2019). Despite the great advances in understanding the ecological, evolutionary, physiological, biochemical, and molecular aspects involved in desiccation tolerance of plants, the genetic dynamics which underpin desiccation tolerance are still a black box (Dinakar and Bartels, 2013; Giarola et al., 2017).

**FIGURE 1:**
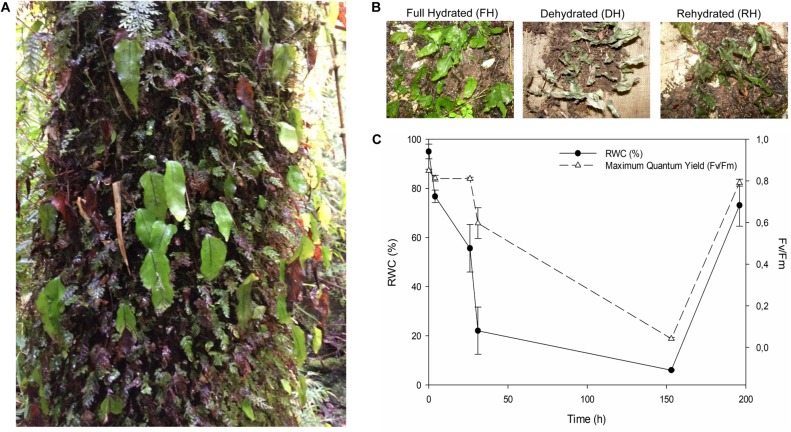
**(A)** *Hymenoglossum cruentum* in their natural environment over the base of the trunk of a host tree. **(B)** Appearance of *H. cruentum* during the experimental desiccation-rehydration process. **(C)** Monitoring of the changes in relative water content (RWC) and photochemical efficiency (*F*_v_/*F*_m_) during the desiccation-rehydration process. Each parameter was evaluated on three biological replicates (detached fronds for RWC and attached fronds for *F*_v_/*F*_m_).

Desiccation tolerant species has been described mainly for habitats with seasonal rainfall, extreme variations in moisture availability, and long dry periods (Bartels and Salamini, 2001; Alpert, 2006). Surprisingly, some of them also inhabit humid ecosystems such as *Lindernia brevidens* from the tropical rain forests (Proctor, 2003; Phillips et al., 2008) or the epiphytic ferns of the Hymenophyllaceae family (Pteridophyta) from temperate rain forest (Proctor, 2003). These last, are poikilohydric species, commonly called filmy ferns because they possess membranous fronds of a single layer of cells; have very thin or absent cuticles; present no differentiated epidermis; and do not possess a real mesophyll nor stomata (Proctor, 2012; Saldaña et al., 2014; Fallard et al., 2018). They show a remarkable resilience to fast and frequent cycles of desiccation-rehydration, which occurs especially in the summer period. These ferns are homoiochlorophyllous, maintaining high content of photosynthetic pigments during the desiccated state (Flores-Bavestrello et al., 2016). In the Chilean temperate rain forest, the Hymenophyllaceae family represents an important component of the epiphytic species diversity (17 species; Parra et al., 2012). *Hymenoglossum cruentum* is a highly abundant species that colonizes higher-humidity/low-light microenvironments (Figure 1A). Besides dehydration, this fern must also deal with the occurrence of high intensity sunflecks, particularly frequent on sunny days. This leads to rapid and significant increases in light intensity and the evaporative demand (Chazdon and Pearcy, 1991; Leakey et al., 2005; Coopman et al., 2010; Niinemets et al., 2018). Photochemically, *H. cruentum* is well suited to live in shaded conditions (Parra et al., 2015; Flores-Bavestrello et al., 2016). Previous studies ranging from cellular to ecophysiological approaches have suggested that the DT response of *H. cruentum* is mainly constitutive, and that re-watering induces the most severe oxidative stress (Parra et al., 2015; Flores-Bavestrello et al., 2016; Fallard et al., 2018; Niinemets et al., 2018). However, given the characteristic of their microhabitat plus the high frequency of dehydration-rehydration cycles this fern can experience, the DT mechanism of *H. cruentum* would rely on highly coordinated genetic pathways to protect, maintain, repair and reestablish the cellular integrity and activity.

The objective of this research is to identify genes expressed during desiccation and rehydration in *H. cruentum* to better understand the genetic dynamics behind its desiccation tolerance mechanism. For this purpose, an experimental desiccation-rehydration process on *H. cruentum* was conducted. We performed clustering and network analyses on transcriptome data to identify key genes associated to its capacity to dehydrate and rehydrate. This work will contribute to the very limited information on gene discovery associated to desiccation tolerance in Hymenophyllaceae filmy ferns found in literature (Ostria-Gallardo et al., 2020).

Our results reveal key genes orchestrating a preventive state at full hydrated; strong balance between avoiding cell death and defense when dehydrated, and detoxifying pathways and stabilization of photosystems during rehydration.

## Materials and Methods

### Plant Material and Experimental Design

Intact *H. cruentum* (Cav.) C. Presl. ferns were collected from a second-growth forest located in Katalapi Park, Puerto Montt, Chile (41°31′07.5′′S, 72°45′2.2′′W). The climatic zone corresponds to a coastal temperate evergreen forest (see Saldaña et al., 2014 for further details of the sample site). Branches and barks of trees covered with *H. cruentum* were taken from the base to 1 m of vertical height of the trunks. A field guide was used to identify individuals of *H. cruentum*, and were further corroborated with samples from the existing collection of the Herbarium of the Universidad de Concepción^1^ (Herbario CONC, Departamento de Botánica, Universidad de Concepción, Barrio Universitario s/n, Casilla 160-C, Concepción, Chile). All samples were brought to a shaded experimental nursery garden with automated irrigation and *ca.* 43 μmol of PAR at midday sun, at facilities of Universidad de La Frontera, Temuco. The filmy ferns went through a cycle of desiccation-rehydration (Figure 1B) by adjusting the irrigation system. First, *H. cruentum* ferns were subjected to sprinkler irrigation pulses of 3 min, at intervals of 15 min for 4 h to reach a fully hydrated (FH) state. Immediately, fronds from three different individuals (biological replicates) were collected to quantify the relative water content. Then, the irrigation was stopped for 7 days to induce fronds desiccation (DH) and to monitor their change in relative water content (RWC) and maximum quantum yield (*F*_v_/*F*_m_). Specifically, samplings for RWC were conducted after 3, 24, 48, and 168 h without irrigation, collecting three biological replicates in each case. At the 7th day without irrigation, we resume the irrigation to induce fronds rehydration (RH) during 3 h with the pulses and intervals previously indicated, and immediately fronds from three biological replicates were collected to measure the RWC. In parallel, the *F*_v_/*F*_m_ were measured on attached fronds of three biological replicates during the dehydration-rehydration process described above. In addition, fronds from three biological replicates were collected in liquid nitrogen during the desiccation-rehydration process and stored at −80°C until processed for RNA isolation. Specifically, sampling was made at ca. 100, 80, 6, and 80 %RWC. It is worth mentioning that during the desiccation-rehydration experiment, we sampled fronds at the beginning and at the end of the dehydration, in order to capture early and late transcriptional responses.

### RNA Isolation

The RNA was isolated from the three biological replicates collected at each of the hydration states (see the section above) using Ultra Clean^TM^ Plant RNA Isolation Kit (Mo Bio, Carlsbad, CA, United States) and purified with Total RNA I kit (Omega Bio-Tek, Norcross, GA, United States) according to manufacturer’s instructions. The yield and quality of isolated RNA was checked by 1% agarose gel electrophoresis. The RNA concentration was determined by the *A*_260_/*A*_280_ ratio with an Infinite M200 Nanoquant. The yield and quality of the RNA isolation samples was determined by an Agilent 2100 Bioanalyzer with an RNA integrity number (RIN) of six as cutoff. Then the RNA was precipitated with two volumes of acetate:ethanol solution (1:10 v/v) to avoid degradation and subsequently sent for sequencing to the Center for Integrative Biology at Universidad Mayor, campus Huechuraba, Santiago, Chile.

### RNA-Seq Transcriptome Profiling and Transcript Quantification by RT-qPCR

A total of three libraries, consisting in a pool of the three biological replicates at each hydration state were sequenced in a single lane of an Illumina MiSeq platform (Illumina Inc., San Diego, CA, United States) for 150 cycles in paired-end mode. The reads were processed with NGSQC Toolkit v2.3^2^ (Patel and Jain, 2012) based on their Phred score (*Q*-score) for quality filtering. High quality filtered reads were assembled *de novo* by using the Trinity software package v2.1.1 (Grabherr et al., 2011; Haas et al., 2013). Transcriptome assembly, reads mapping and transcript annotation were performed at the Troquil Linux cluster at Centro de Modelación y Computación Científica (CMCC, Universidad de La Frontera) using 12 processors Intel Xeon E5-4640 and 192 GB of shared memory. Assembly completeness was quantified by comparing the transcripts to a set of highly conserved single-copy orthologs. This was accomplished using the BUSCO (Benchmarking Universal Single-Copy Orthologs) pipeline v3 (Simão et al., 2015) compared to a predefined set of 1440 Embryophyta single-copy orthologs from the OrthoDB v9.1 database (Zdobnov et al., 2016). The RNA-seq by expectation maximization (RSEM) was used to estimate the abundance of transcripts for the FPKM value (Fragments per kilobase per transcript per million mapped reads) on each sample (Li and Dewey, 2011; Zhang et al., 2018). The resulting RSEM-estimated gene abundances for each hydration state were merged into a matrix to determine differentially expressed genes (DEGs) with the run_DE_analysis.pl script from Trinity, which involves the Bioconductor package edgeR in R statistical environment (Robinson and Oshlack, 2010; R Core Team, 2013). The transcriptome was aligned into the SwissProt database using BLAST+. Functional annotation was performed using PANTHER^3^.

From the DEGs data, we selected candidate genes that would be pivotal in the desiccation tolerance response of *H. cruentum* for quantification by RT-qPCR. cDNA was obtained from total RNA isolated from three biological replicates from each hydration state by using the AffinityScript RT-qPCR kit according to manufacturer’s instructions (Stratagene, Cedar Creek, TX, United States). For mRNA levels normalization, five housekeeping genes were evaluated as references genes using the geNorm software, by calculating the stability measure “M” of the expression of the potential reference gene, as the average pair-wise variation compared with other tested genes (Vandesompele et al., 2002). Those genes with an *M* < 1.5 present the most stable expression level (Supplementary Figure S1). Hence, the *thylakoid lumenal protein* (*thy*), *protein DJ-1 like* (*DJ*), and *elongation factor 1 alpha* (*EEF1A1*) were then used as reference genes. The expression of these genes was stable among frond’s hydration states. Total RT-qPCR reaction volume was 20 μl, containing μl of cDNA template and 10 μM of primers. The reaction was carried out using a Stratagene Mx3000p under the following conditions: 95°C for 10 min, followed by 40 cycles of 95°C, 15 s; 60°C, 15 s; 72°C, 15 s. Data analysis was performed by Stratagene MxPro. Significant differences of gene expression were determined by an ANOVA test with *P*-value ≤ 0.05.

### Self-Organizing Maps (SOM) Analysis

Normalized RSEM-estimated counts of *H. cruentum* that met the expression values determined from the model described above were used for the clustering method (as described in Chitwood et al., 2013; Ostria-Gallardo et al., 2016). Specifically, only genes that vary significantly in expression across fronds hydration states were analyzed. We used custom R scripts^4^ to detect the effects of fronds hydration state on gene expression. We selected transcripts from the upper 50% quartile of coefficient of variation for expression across hydration states (33,633 transcripts total). The scaled expression values within samples were used to cluster these genes for a multidimensional 2 × 3 hexagonal Self-Organizing Maps (hereafter SOM) using the Kohonen package on R (Kohonen et al., 1997; Wehrens and Buydens, 2007). Hundred training interactions were used during clustering with a decrease in the alpha learning rate from ca. 0.0015 to 0.0009. SOM outcome was visualized into pie charts for codebook vectors to obtain the counts number and mean distance of the genes assigned to each Node (Wehrens and Buydens, 2007). The box plot option from the ggplot2 package on R was used to visualize the gene accumulation patterns associated to the hydration states of the fronds in each Node. Finally, the genes of each Node were analyzed for GO enrichments terms at a 0.05 false discovery rate cutoff. This first clustering process was a necessary step to identify groups of genes highly associated to frond’s water status according to the experimental design.

### Gene Co-expression Network Analysis

In order to further explore the interaction of the selected candidates DEGs quantified by RT-qPCR, and the gene clusters generated by the SOM analysis, we used a Gene Regulatory Network-based (GRN) approach to study the interactions between gene expression at each hydration state of fronds, following the workflow of Ostria-Gallardo et al. (2018). Specifically, from the SOM clustering method, we selected a subset of 26,905 transcripts showing the highest accumulation pattern for a given frond hydration state (Supplementary Dataset S1). From these transcripts, the normalized count values of annotated genes (157 genes total) were selected to construct a weighted gene co-expression network by using the Weighted Gene Coexpression Analysis (WGCNA; Langfelder and Horvath, 2008) R package. The soft threshold power (β) value chosen was 9 to meet the scale-free topology criteria. An adjacency matrix was calculated and transformed into a topological overlap matrix. Afterward, we applied the resources and algorithms of the graph generator and community structure functions of the igraph R package (Csardi and Nepusz, 2006) to explore network properties such as connectivity, centralization, modularity, and community structure. Finally, we utilized custom graph functions of the igraph package for network visualization.

### Sequence Submission

The quality-filtered, barcode-sorted, and trimmed short read data set used for transcriptome assembly and gene expression analysis, was deposited in the NCBI Sequence Read Archive (SRA) under accession SRR9056108, SRR9056827, and SRR9056841.

## Results

### Changes in Relative Water Content and Maximum Quantum Efficiency of PSII During a Desiccation-Rehydration Cycle

During the first 4 h after cessation of irrigation (beginning of dehydration), *H. cruentum* dehydrated reaching 80% of RWC. After 26 h without irrigation, the fronds showed a RWC of 55% (Figure 1C). During this period of dehydration *F*_v_/*F*_m_ remains stable at values of ∼0.8. When fronds reach ca. 20% of RWC after 30 h without irrigation, the values of *F*_v_/*F*_m_ started to decay near to 0.6. After a week without irrigation, the frond’s RWC and *F*_v_/*F*_m_ decayed to ca. 6% and 0.04, respectively. When irrigation was reestablished at the end of the experiment, *H. cruentum* had a faster rehydration and recovery of *F*_v_/*F*_m_, reaching values of ca. 73% and 0.8 in RWC and *F*_v_/*F*_m_, respectively.

### *De novo* Assembly, Completeness Assessment, and Transcriptome Annotation

The sequencing of *H. cruentum* libraries resulted in a total of 12,596,956 pair-end reads, of which 31.17% corresponded to full hydrated state (FH), 34.79% to desiccated state (DH), and 33.04% to the rehydrated state (RH) (Supplementary Dataset S2). After quality filtering process, the reads were assembled into 75,159 isotigs ranging from 201 to 3,800 bp with a mean length of 697 bp (s.d. 796 bp and a median of 353 bp; Supplementary Figure S2A) distributed in 59,556 isogroups. The completeness assessment by BUSCO showed that 66.5% of conserved orthologs were present, of which 47.8% were single copy and 18.7% were present as duplicate. By including fragmented genes, the final gene recovery percentage increases to 71.2%, with 28.8% of genes of embryophyte set missing in the final *H. cruentum* transcriptome.

Based on the BLAST hit of annotated sequences, the taxonomic distribution of top hits showed that most common organism are mosses (22%, *Physcomitrella patens* and *Selaginella moellendorffii*) and angiosperms (16%, *Picea sitchensis* and *Amborella trichopoda*) (Supplementary Figure S2B). The functional annotation resulted in 97,258 (52.48%), 42,858 (21.48%), and 39,815 (23.13%) annotated counts for GO categories distributed in biological process (BP), molecular function (MF), and cellular component (CC) (Supplementary Figure S2C). Specifically, the processes with high number of sequences within each GO categories were: metabolic process in BP; catalytic activity and binding in MF; cell part and organelle in CC (Supplementary Figure S2C). Additionally, 12,963 ORFs were annotated as enzymes, with transferases being the most abundant class, followed by hydrolases and oxidoreductases (Supplementary Dataset S3).

### Transcript Expression Patterns Across Dehydration-Rehydration Cycle

**FIGURE 2:**
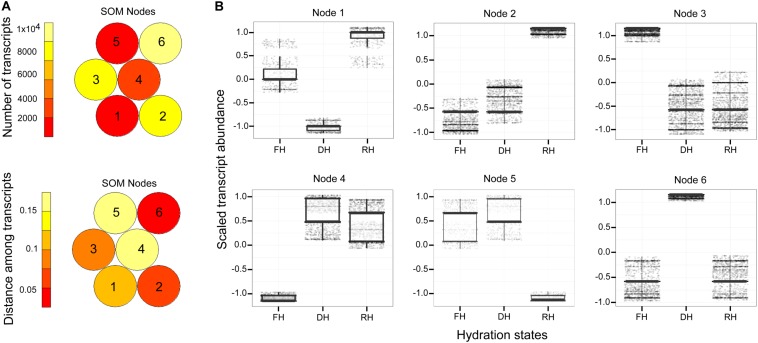
Mapping quality of the constructed self-organizing map (SOM) with a 2 × 3 hexagonal topology applied to *H. cruentum* DE transcripts. **(A)** The heatmaps shows the number of genes (up) assigned to each SOM Node (numbered from 1 to 6) and the mean Euclidean distance of genes (down) mapped within the particular Nodes. Red-like color indicates low count and distance, whereas light yellow indicates high count and distance. **(B)** From the total six Nodes defined after SOM, boxplots were used to visualize and select those Nodes describing the highest pattern of differentially expressed genes for a given hydration state (see details in the “Result” section). For each boxplot, horizontal line represents the median, and bars represent the maximum and minimum values of the scaled gene abundance. *X*-axis labels read as follow. FH, full hydration; DH, dehydration; and RH, rehydration.

**FIGURE 3:**
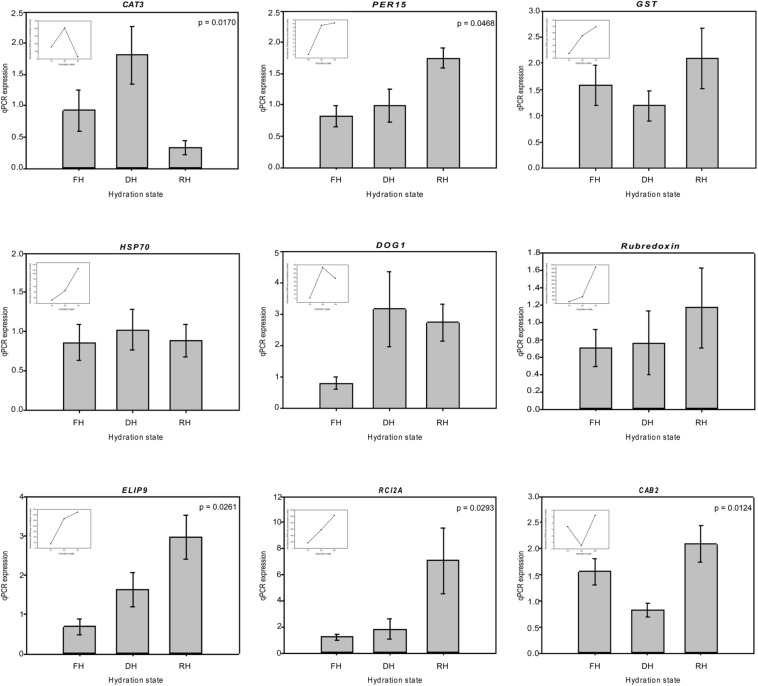
Comparison of the change in expression during the desiccation-rehydration process of candidate genes that would be pivotal in the desiccation tolerance response of *H. cruentum* by quantification of real-time PCR. The inserted panels show the *in silico* RNAseq relative abundance patterns of each gene in a given hydration state. Data are the means of three biological replicates. The value *p*-value is shown for those genes showing significant differences at *p* < 0.05, based on the non-parametric analysis of Kruskal–Wallis.

**FIGURE 4:**
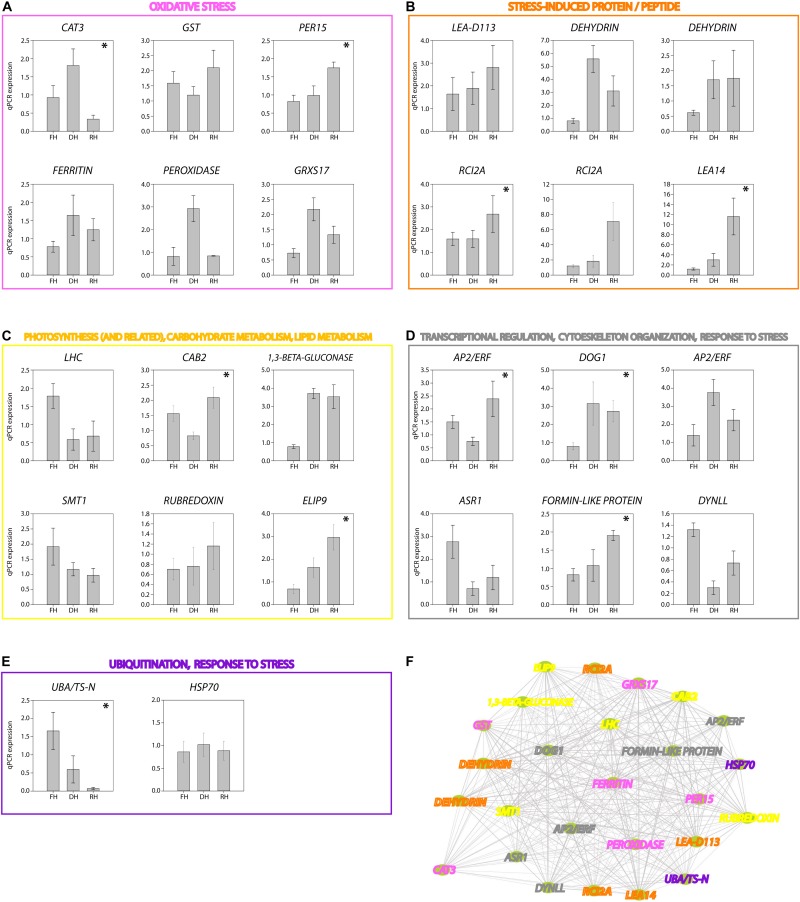
RT-qPCR expression of the 26 transcripts identified as key genes associated to the desiccation tolerance response of *H. cruentum.* Genes are grouped according to their function as follow: **(A)** oxidative Stress (magenta); **(B)** stress-induced protein/peptide (orange); **(C)** photosynthesis, carbon and lipid metabolism (yellow); **(D)** transcriptional regulation, cytoskeleton organization, response to stress (gray); **(E)** ubiquitination, response to stress (purple). Significant differences in expression among hydration states are indicated by the asterisk (*p*-value < 0.05). Panel **(F)** show the connectivity among the 26 genes. The colors of the gene names correspond to their function indicated in panels **(A–E)**. Please see the main text for discussion of the function and co-expression of these genes regarding the hydration state of the frond of *H. cruentum.*

A major focus of this study was to identify genes and better understand the dynamics of their expression associated with the level of frond’s hydration of *H. cruentum*. The self-organizing map (SOM) clustering analysis was used to depict transcripts with similar accumulation patterns related to the hydration state (Figure 2). Based on the topology reached after the iteration process, the SOM nodes 2, 3, and 6 showed the higher number of transcripts and also the lower mean distance among them (Figure 2A). Regarding the transcript accumulation on each frond’s hydration state (Figure 2B), node 3 showed higher accumulation of transcripts associated to the Full Hydrated state, with a GO enrichment reflecting an active metabolic activity related to lipid transport, photosynthesis, transcription, and flavonoid glucuronidation (Supplementary Dataset S4). Node 6 showed higher accumulation pattern of transcripts in the dehydrated state which was enriched in genes involved in defense response, antioxidant, cell division, lipid metabolic process, heat acclimation, homeostasis of membrane potential (Supplementary Dataset S4). Finally, node 2 included higher accumulation of transcripts in the rehydrated state, showing an enrichment of genes associated with protein-chromophore linkage, lipid biosynthesis, hormones responses, cell redox homeostasis, response to oxidative stress, glutathione metabolic process, tricarboxylic acid cycle, protein glutathionylation, and membrane potential homeostasis (Supplementary Dataset S4). From the accumulation patterns of transcripts observed in the SOM analysis, we identify and quantify the expression of 26 candidates DEGs potentially key for the desiccation tolerance response of *H. cruentum* (Supplementary Dataset S5). As a reliability control, we compared the qPCR expression and the *in silico* expression of some of these genes (*CATALASE-3*, *CAT3*; *PEROXIDASE 15*, *PER15*; *GLUTATHIONE S-TRANSFERASE*, *GST*; HEAT SHOCK PROTEIN *70*, *HSP70*; *DELEY OF GERMINATION 1*, *DOG1*; *RUBREDOXIN*, *EARLY LIGHT INDUCIBLE PROTEIN 9*, *ELIP9*; *RARE COLD INDUCIBLE 2A*, *RCI2A; CHLOROPHYLL A BINDING PROTEIN 2*, *CAB2*) (Figure 3). The 26 identified genes are grouped into the following functions: oxidative stress; stress-induced protein/peptide; photosynthesis, carbohydrate and lipid metabolism; transcriptional regulation, cytoskeleton organization; ubiquitination and response to stress (Figures 4A–E). Further, the expression patterns of 10 of these genes (namely *CAT3*, *PER15*, *RCI2A*, *LEA14*, *CAB2*, *ELIP9*, *AP2/ERF*, *DOG1*, *FORMIN-LIKE PROTEIN*, and *UBA/TS-N*) varied significantly along the hydration state of *H. cruentum*, suggesting a pivotal role in the desiccation-rehydration response of this filmy fern.

### Gene Co-expression Network of *H. cruentum* in Response to Desiccation-Rehydration Cycle

**FIGURE 5:**
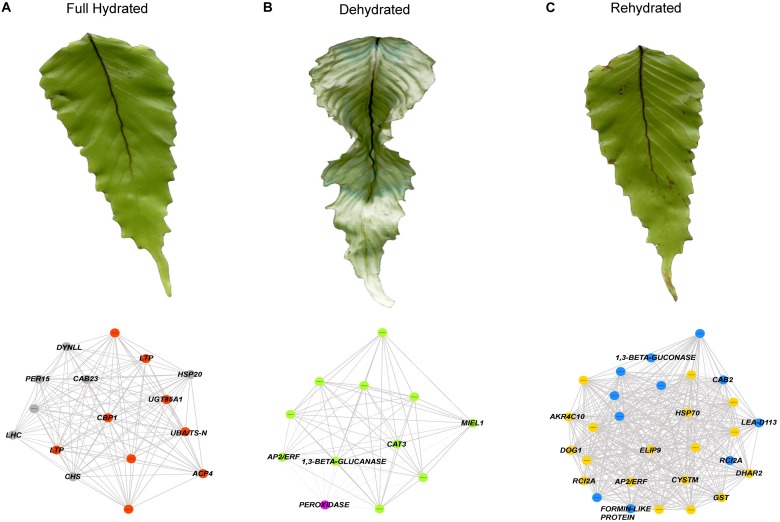
Gene co-expression networks analysis for **(A)** the full hydrated (FH), **(B)** dehydrated (DH), and **(C)** rehydrated (RH) states of *H. cruentum* fronds. The genes used for the network construction were obtained from Nodes 2, 3, and 6 (see Figure 2B). Each network included two modules indicated by the colors of the circles (gray and orange for FH, green and magenta for DH, blue and yellow for RH). Each of the modules contain transcripts with denser connections representing predicted interactions. The names of those genes showing higher connections within a given hydration state are indicated and their specific functions are discussed in the text.

Based only on the identified genes, we studied the interconnections of the 26 tested transcripts (Figure 4F). The resultant network reveals candidates for hub genes (i.e., genes with high connectivity and interaction with other genes). For example, *DOG1*, *FERRITIN*, *AP2/ERF*, *CAT3*, *FORMIN-LIKE PROTEIN* appears as highly connected genes in the co-expression network. Then, from the annotated genes (including the 26 tested genes) showing the higher accumulation patterns in SOM nodes associated to the full hydrated, desiccated and rehydrated states, we construct a weighted gene coexpression networks for each of the hydration states (Figures 5A–C; see section “Materials and Methods” for details). Based on the Fast Greedy modularity optimization algorithm for finding community structure, all of the constructed gene-coexpression networks had two modules. An overview of the resultant networks for each hydration state showed that, at full hydration (Figure 5A), the co-expressed genes were involved in: light harvesting complexes and reaction centers of photosystems I and II (e.g., *LHC* and *CAB23*); intracellular motility (*DYNLL*); protective system against oxidative stress (e.g., *PER15*); and protein turn-over (*UBA/TS-N*). Under desiccation (Figure 5B), the resultant network showed low interconnection of genes, underlying, however, the function of apoplastic detox of ROS (*CAT3*) and regulation of plant defense and cell death (*AP2/ERF* and *MIEL1*). Finally, the resultant network from the rehydrated state (Figure 5C) showed the highest number of co-expressed genes. The main processes represented by these genes are: cytoskeleton organization (*FORMIN-LIKE* PROTEIN), protein protection (*LEA-D113*), transcriptional regulation of seed dormancy (*DOG1*), antioxidant and redox homeostasis (*GST* and *DHAR2*), photosystem II accumulation (*CAB2*), carbohydrate metabolism and desiccation tolerance (*1*,*3-β-GLUCONASE* and *GRD1*), and stress tolerance perception and signaling (AP2/ERF and *CYSTM*). Also, genes involved, lignin biosynthetic pathway (*CSE*), and detoxification of sugar-derived carbonyls (*AKR4C10*).

## Discussion

Most of the studies describing the desiccation tolerance response have focused on plants that dehydrate and rehydrate slowly (Giarola and Bartels, 2015; Giarola et al., 2016; Liu et al., 2018). Most of these plants are poikilochlorophyllous, which present major loss of photosynthetic pigments, which contributes to diminish the risk of over-energization and reactive oxygen species (ROS) production in desiccated leaves. On the other hand, there are few studies on fast desiccating-rehydrating homoiochlorophyllous species, mainly in Bryophytes (Gao et al., 2017) few angiosperm (Deng et al., 2003; Charuvi et al., 2015, 2019) and species from Hymenophyllaceae (Proctor, 2012; Niinemets et al., 2018). Previous studies have described and characterized several responses of *H. cruentum* to the desiccation and rehydration process (Garcés et al., 2015; Parra et al., 2015; Bravo et al., 2016; Flores-Bavestrello et al., 2016; Fallard et al., 2018; Niinemets et al., 2018). However, the genetic basis and transcriptional dynamics behind the DT response along the hydration states of *H. cruentum* fronds have never been addressed before our present study. This becomes relevant considering that RNA molecules (particularly mRNA) are the way in which cells carry out the instructions encoded in the DNA. To our knowledge, there is very limited information about the transcriptional dynamic associated to dehydration-rehydration in Hymenophyllaceae species (Ostria-Gallardo et al., 2020). Thus, one of our goals was to better understand the genetic dynamics behind the mechanisms involved in the desiccation-rehydration kinetic of *H. cruentum*.

Our *de novo* transcriptome assembly revealed few transcripts showing – *in silico* – differential expression, supporting the idea from proteomic analyses that the desiccation tolerance response of *H. cruentum* would rely mainly on constitutive mechanisms rather than on desiccation-induced ones (Garcés et al., 2015). However, *de novo* protein synthesis must be needed for a complete recovery from the after-stress effects (Niinemets et al., 2018). Identifying patterns in massive gene expression datasets consistent with a biological function is always a challenge, especially in non-model species without a reference genome. Here, by combining the SOM analysis with the best features of the WGCNA and the igraph algorithms, we achieved a powerful data mining procedure (see features and uses in Wehrens and Buydens, 2007; Clark and Ma’ayan, 2011; Ranjan et al., 2015; Ostria-Gallardo et al., 2018). Through the differential gene expression analysis, we found transcripts that serves as candidates for pivotal functions in the DT response of *H. cruentum*. Further quantification of the expression by qPCR of some of them, and the analysis of these genes plus the *in silico* highly abundant transcripts by artificial neural networks tools allowed us to depict clustering patterns of gene expression across the hydration states of *H. cruentum* and decipher key mechanism used by this fern to tolerate desiccation.

The functional annotation of genes associated to the full hydration state reflect an active metabolic activity. We found high abundance of specific transcripts involved in intracellular motility, antioxidant activity, protein turnover, plant defense and stabilization of macromolecules (e.g., *DYNLL*, *PER15*, *UBA/TS-N*, *ASRP1*, *SMT1*, and *HSP20*). All of these highly abundant transcripts, and particularly *PER15* and *UBA/TS-N*, indicate that *H. cruentum* would presents a line of response and defense to prevent deleterious effects under an eventual dehydration process. This may have been shaped by the microenvironmental dynamics of their niche, given the increased frequency of sunflecks accompanying dehydration events, which induces rapid and local changes from low-light/high humidity to high-light/low humidity (Coopman et al., 2010; Niinemets et al., 2018). Interestingly, the catalytic activity of STM1 serves as the starting point in sterol biosynthesis. Evidence is growing about the regulatory roles of sterols in plant development, hormone synthesis and response to environmental stimulus (Schrick et al., 2004; Men et al., 2008; Carland et al., 2010). The expression of the *SMT1* gene indicates the use of the 24-methylene- cycloartenol pathway for phytosterol biosynthesis, and would be key for the sensitivity of *H. cruentum* to perceive and trigger a signal transduction in response to dehydration (Valitova et al., 2019).

During dehydration and particularly important at desiccated stage, the absorbed energy exceeds the capacity of photosystems to use the energy of light to photochemistry (Anjum et al., 2016), mainly due to an overreduction of PSII electron acceptors. In our experiment, there was a significant decrease of frond *F*_v_/*F*_m_ during the dehydration and desiccation stage. It is known that during the desiccated state, *H. cruentum* re-organizes its photosynthetic apparatus into an energy quencher that dissipates the absorbed energy through the constitutive pathway for non-photochemical quenching (ΦNO) (Flores-Bavestrello et al., 2016). The glycosyltransferase function of *UGT85A1* (present in the SOM but not quantified by RT-qPCR) particularly over the *trans*-zeatin homeostasis, may also contribute to energy dissipation under the initial phase of dehydration, since have been associated to an increased stress tolerance to heavy metals, drought, and salt stress (Nishiyama et al., 2011; Jin et al., 2013; Li et al., 2015). However, these thermal dissipation mechanisms by itself cannot withstand several days of desiccation, and the over-energization of the photosynthetic apparatus plus the increase of ROS can damages severely the thylakoid membranes and proteins during dehydration (Niinemets et al., 2018). Interestingly, we observed high transcript abundance of the *CAT3* and *RUBREDOXIN* genes (both validated by RT-qPCR). Both were identified as a key desiccation related-genes by the co-expression network analysis (Figure 5B). These results highlight two ROS scavenging mechanisms used by *H. cruentum* when desiccated. The first is the role that the peroxisome and the extracellular region could have because of the increase of *PEROXIDASE* and *CAT3*, respectively. Peroxisomes are both major sources of ROS production and degradation, and the site of important anti-oxidant defense (Mhandi et al., 2010; Nordgren and Fransen, 2014). Specifically, the apoplastic *CAT3*, and the *PEROXIDASE* genes showed a significant increase in expression during desiccation (Figure 4), indicating an antioxidant capacity and ROS alleviation that would be accumulating in the apoplastic region or mobilized into the peroxisomes (Calderon et al., 2013; Clemente-Moreno et al., 2019a). The other mechanism involves the increasing abundance of transcripts of a *RUBREDOXIN* gene, which encode a chloroplast-localized protein and inhibit the accumulation of hydrogen peroxide (H_2_O_2_), playing a key role in the maintenance of chloroplast structures and thylakoids stabilization during both, desiccation and rehydration to diminish the photo-oxidative damage (Gechev et al., 2012; Li et al., 2016). In addition, despite the extremely low water content and photosynthetic activity, we found an enrichment of GO categories related with organelle, metabolic processes, and catalytic activity (Supplementary Figure S2C). When coupled to the results of gene expression, our results strongly suggest that *H. cruentum* do not enter into a complete metabolic arrestment when desiccated. Rather, it maintains active mechanisms that enhances antioxidant and defense pathways (e.g., *CAT3*, Figures 4, 5), and in parallel, turn-off the cell death program through the ubiquitination machinery (as suggested by the *in silico* abundance of the *MIEL1* transcript, see Lin et al., 2019; An et al., 2020). This is consistent with the fast recovery of the photosynthetic activity observed when fronds get rehydrated (Figure 1C), and their homoiochlorophyllus strategy (Flores-Bavestrello et al., 2016). Therefore, our data strongly support the hypothesis that species from Hymenophyllaceae are partially desiccation tolerant (Fallard et al., 2018).

Contrary to what was expected, we did not find a significant increment of ELIP genes during desiccation instead we find an increment during rehydration (Figure 4C). Accordingly, it seems that the higher ROS increase in *H. cruentum* is during rehydration. In general, Hymenophyllacea species have fronds with high hydrophilicity and capillarity, resulting in rapid rehydration (Niinemets et al., 2018 and references therein). It has been reported for Hymenophyllaceae species, that during rehydration there is a major emission burst of the lipoxygenase pathway volatiles (LOX), which serve as markers for stress severity (Niinemets et al., 2018), suggesting a rapid increase of ROS after rewatering. Hence, counterintuitively, the rehydration phase supposes the most severe stress condition. According to *in silico* our results, a higher proportion of DE genes occurs during rehydration (Figures 2A,B). Also, the RT-qPCR plus the SOM output, and the network analysis showed the highest abundance and number of co-expressed genes at the rehydrated state (Supplementary Dataset S5 and Figures 4, 5C). At rehydration, there is: an increase in transcript abundance of genes involved in photoprotection (*ELIP9*); regulation of membrane permeability and fluidity mediated by the significant high expression of the *RCI2A* gene at the rehydrated state (Figure 4); and the co-expression of genes encoding for regulation of seed desiccation tolerance (*DOG1*; Bryant et al., 2019) and molecular stabilizers (*HSP70*) (Figures 4, 5C). Besides these key genes, we also observed an enrichment in components of the glutathione branch of the Foyer–Halliwell–Asada cycle (e.g., *GRXS17*, *GST*; Foyer and Noctor, 2011), pointing out the role of sulfur metabolism in detoxifying ROS (Clemente-Moreno et al., 2019b). Finally, a high abundance of transcripts related to photosystem stabilization (*CAB2*) and glucans of the non-cellulosic matrix (*1*,*3-*β*-gluconase*) would be key for the recovery from the desiccated state. We propose that the function of these genes in *H. cruentum* fronds would be related to a high oxidative pressure from the desiccated state plus mechanical and chemical changes of the cell wall during the unfolding of rehydrating fronds, reflecting a tight regulation of scavenging oxidative stress while reestablishing membrane and cell wall integrity, and the photosynthetic function during rehydration.

In summary, our study identified key genes during the transcriptional dynamics along the desiccation-rehydration cycle of *H. cruentum*. There is strong genetic evidence to extend our understanding of the underlying processes involved in the desiccation tolerance response of this epiphytic filmy fern. Thus, at full hydration state *H. cruentum* is well prepared to sense, protect and reorganize their cellular structures to an eventually massive loss of water. Subsequently during dehydration and desiccation, they sustain a basal metabolism destined to deal with the oxidative stress over photosynthetic pigments in parallel with cell death avoidance. Finally, upon rehydration, which is likely to be the most stressful condition, there is: an increase of co-expressed genes combining mechanisms to cope with the ROS accumulation from the desiccated state; the expression of seed-related genes involved in the acquisition of desiccation tolerance with photoprotective genes, stabilization of photosynthetic membranes and proteins, and favor cell wall expansion. The desiccation tolerance response of *H. cruentum* underpin the rapid recovery during rehydration and would be an integration of the microenvironmental dynamics of their niche, given the frequency of sunflecks that induces rapid and local changes from low-light/high humidity to high-light/low humidity.

Our study provides novel insights into understanding the recovery mechanisms of this homoiochlorophyllous desiccation tolerant fern species. Also, with our results, new exciting questions arise, such as what would be the roles and contribution of specific molecules (e.g., RNA-binding proteins, non-coding RNAs sequences) and posttranscriptional regulations that may have important roles in the desiccation tolerance strategy. Also, much more is needed to know how similar -or not- are the gene-coexpression networks behind the DT mechanisms of other co-occurring species from Hymenophyllaceae, given the extraordinary variety in niche preferences and microclimatic conditions that different species encounters along a host tree. Further works combining physiological, metabolomic, genomic, and evolutionary-developmental approaches are needed to achieve a comprehensive understanding of the desiccation tolerance response in homoiochlorophyllous species with fast and frequent desiccation-rehydration cycles. This seems to be the most viable strategy to look into for target genes for biotechnological use to prevent the impacts of extreme drought events on crops rather than on extensive structural modification and recovery needed to reach poikilohydricity.

## Data Availability Statement

The datasets generated for this study can be found in the Sequence Read Archive (SRA) under accession SRR9056108, SRR9056827, and SRR9056841.

## Author Contributions

GL, GB, AG-M, and LB conceived the study. AG-M and LB provided materials. GB, AF, and LB coordinated sampling and desiccation-rehydration experiments. GB and AF isolated the RNA. PM conducted and supervised the sequencing. GL assembled *de novo* the transcriptomes. GL and EO-G analyzed the transcriptomic data sets. EO-G, LB-G, and IE worked on interpretation of transcriptomic data. EO-G and LB-G generated and analyzed the cluster and artificial networks. EO-G, LB, and GL wrote the article with contribution of all other authors. All authors read, edited, and approved the article.

## Conflict of Interest

The authors declare that the research was conducted in the absence of any commercial or financial relationships that could be construed as a potential conflict of interest.

## Footnotes

1 http://www2.udec.cl/~herbconc/index.htm

2 http://www.nipgr.res.in/ngsqctoolkit.html

3 www.pantherdb.org

4 https://github.com/eostria/H.-cruentum-Gene-Co-expression-Network

## Supplementary Material

FIGURE S1 Stability ranking of potential candidate genes for selection of housekeeping genes used as endogenous control in the RT-qPCR analysis. Values of the stability parameter M < 1.5 reflect high expression stability.

FIGURE S2 **(A)** Top-hit species distribution of the final transcriptome of *H. cruentum* showing abundance of top hits to sequences of Bryophyta, Lycophyta, and Pinophyta. **(B)** Transcript size distribution showing high proportion of small transcripts in the final transcriptome assembly. **(C)** GO-category distribution of *H. cruentum* transcripts among level 2 GO categories: Cellular component (CC), biological process (BP) and molecular function (MF). Values indicated on X axis are not in the same scale.

DATASET S1 Selected transcripts from the SOM analysis with the highest accumulation patterns associated to a particular hydration state of the fronds. Column A show the transcript ID. Rows 2 to 142 show the annotation of some of these transcripts. From row 143 onwards, Columns B to N show the expression patterns and statistics used as input for the SOM analysis. Column O indicate to which Node belong each transcript. Column P indicate the neighborhood distance of a transcript with all other transcript within the Node.

DATASET S2 The table shows the number of reads before and after the quality filtering process of the *Hymenoglossum cruentum* transcriptome assembly. In addition, the total percentage of identified reads and the percentage of reads correlated with the hydration state of *H. cruentum* fronds are shown.

DATASET S3 Output of transcript annotation and Gene Ontology (GO) categories. Sheets 2 to 4 indicates the GO for Molecular Function, Cellular Component, and Biological Process, respectively.

DATASET S4 Datasheet with the ID and GO enrichment of the transcripts from selected Nodes used for further analysis of RT-qPCR and gene co-expression networks.

DATASET S5 List of the identified and selected transcripts from the *in silico* dataset that were quantified by RT-qPCR. The table indicates the transcript ID, the name of the gene and its function.
