# Supplementary figures and images for: Decoding Gene Networks Modules That Explain the Recovery of Hymenoglossum cruentum Cav. After Extreme Desiccation
Source: Front Plant Sci. 2020 May 15;11:574. doi: 10.3389/fpls.2020.00574 (PMC7243127; doi:10.3389/fpls.2020.00574)

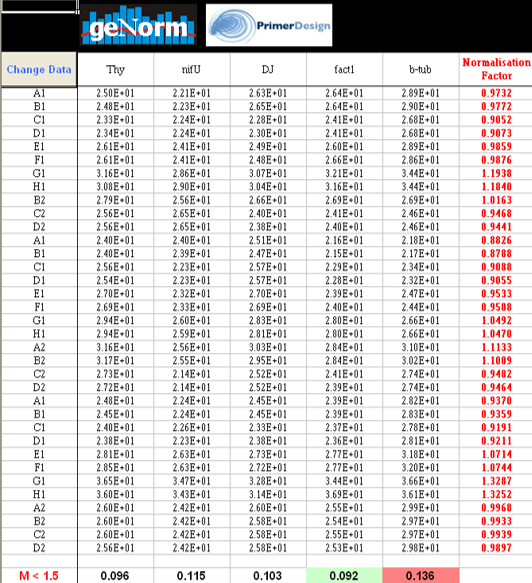

Supplement: FIGURE S1 — Stability ranking of potential candidate genes for selection of housekeeping genes used as endogenous control in the RT-qPCR analysis. Values of the stability parameter M < 1.5 reflect high expression stability. [file Image_1.TIF]
