# Supplementary material for: Decoding Gene Networks Modules That Explain the Recovery of Hymenoglossum cruentum Cav. After Extreme Desiccation
Source: Front Plant Sci. 2020 May 15;11:574. doi: 10.3389/fpls.2020.00574 (PMC7243127; doi:10.3389/fpls.2020.00574)
Supplement: FIGURE S2 — (A) Top-hit species distribution of the final transcriptome of H. cruentum showing abundance of top hits to sequences of Bryophyta, Lycophyta, and Pinophyta. (B) Transcript size distribution showing high proportion of small transcripts in the final transcriptome assembly. (C) GO-category distribution of H. cruentum transcripts among level 2 GO categories: Cellular component (CC), biological process (BP) and molecular function (MF). Values indicated on X axis are not in the same scale. [file Image_2.pdf]

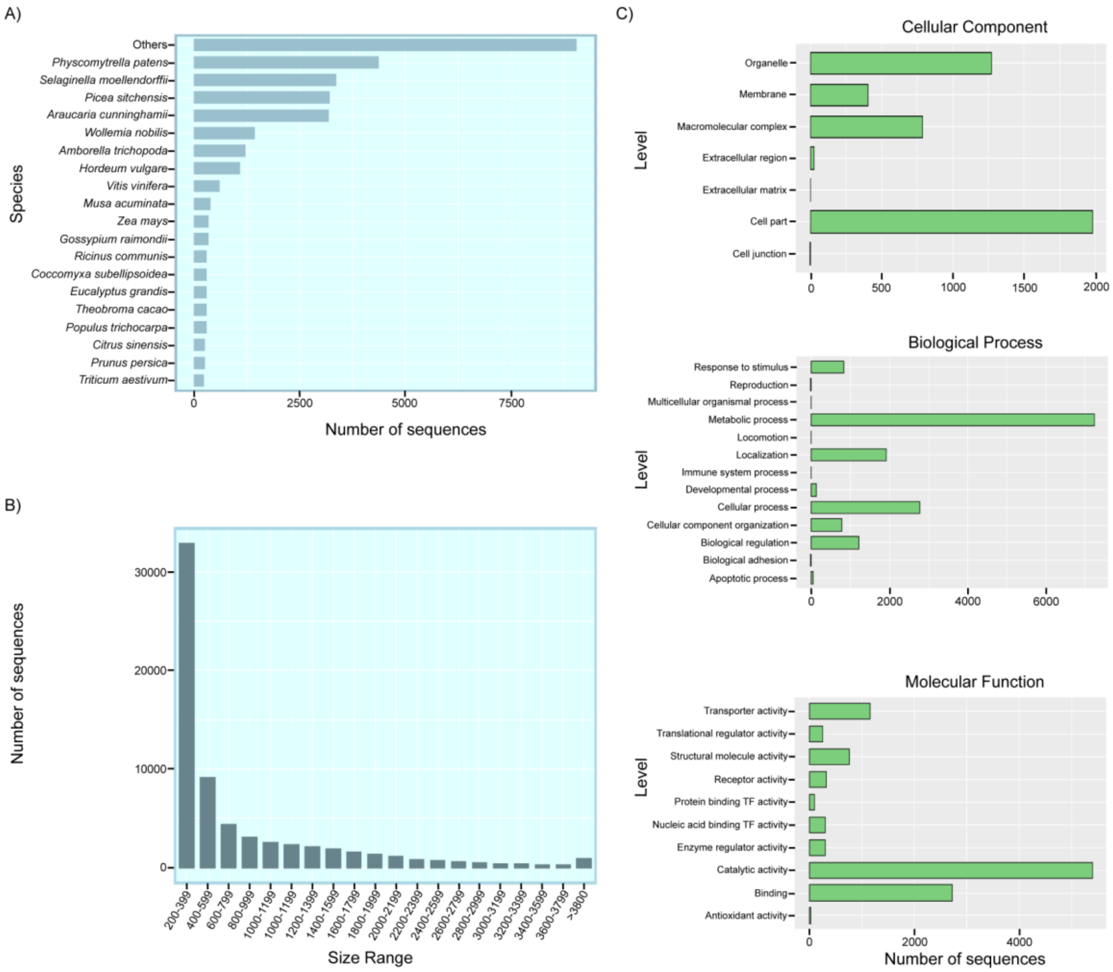

Figure S2. (A) Top-hit species distribution of the final transcriptome of *H. cruentum* showing abundance of top hits to sequences of Bryophyta, Lycophyta, and Pinophyta. (B) Transcript size distribution showing high proportion of small transcripts in the final transcriptome assembly. (C) GO-category distribution of H. cruentum transcripts among level 2 GO categories: Cellular component (CC), biological process (BP) and molecular function (MF). Values indicated on X axis are not in the same scale.
