# Supplementary material for: Decoding Gene Networks Modules That Explain the Recovery of Hymenoglossum cruentum Cav. After Extreme Desiccation
Source: Front Plant Sci. 2020 May 15;11:574. doi: 10.3389/fpls.2020.00574 (PMC7243127; doi:10.3389/fpls.2020.00574)
Supplement: DATASET S2 — The table shows the number of reads before and after the quality filtering process of the Hymenoglossum cruentum transcriptome assembly. In addition, the total percentage of identified reads and the percentage of reads correlated with the hydration state of H. cruentum fronds are shown. [file Data_Sheet_2.DOCX]

**Supplemental Dataset S2.** The table show the number of reads before and after the quality filtering process of the *Hymenoglossum cruentum* transcriptome assembly. In addition, the total percentage of identified reads and the percentage of reads correlated with the hydration state of *H. cruentum* fronds are shown.

| **Total Reads** | **Quality filtered reads (PF reads)** | **% of PF identified reads** | **CV** | **% of PF reads for the full hydration state** | **% of PF reads for the desiccation state** | **% of PF reads for the rehydration state** |
| --- | --- | --- | --- | --- | --- | --- |
| 13,984,369 | 12,596,956 | 98.9977 | 0.0548 | 31.1716 | 34.7868 | 33.0392 |
