# Supplementary material for: Decoding Gene Networks Modules That Explain the Recovery of Hymenoglossum cruentum Cav. After Extreme Desiccation
Source: Front Plant Sci. 2020 May 15;11:574. doi: 10.3389/fpls.2020.00574 (PMC7243127; doi:10.3389/fpls.2020.00574)
Supplement: DATASET S5 — List of the identified and selected transcripts from the in silico dataset that were quantified by RT-qPCR. The table indicates the transcript ID, the name of the gene and its function. [file Data_Sheet_5.DOCX]

| **Identified genes_RT-qPCR validation** | | |
| --- | --- | --- |
| **Number** | **Name** | **Function** |
| Comp15822_c0_seq1 | Catalase | Oxidative stress |
| comp16033_c0_seq1 | Glutathione S-transferase | Oxidative stress |
| comp17594_c0_seq1 | Peroxisoma I catalase | Oxidative stress |
| comp17416_c0_seq1 | Ferritin | Oxidative stress |
| comp20880_c0_seq1 | Formin like proteín | Cytoeskeleton organization |
| comp4045_c0_seq1 | LEA-D113 (group 4A) | Stress-induced hydrophilic protein |
| comp19765_c0_seq1 | Light harvest complex | Photosynthesis |
| comp16080_c0_seq1 | LHC related protein | Photosynthesis |
| comp14833_c0_seq3 | DREB (AP2/ERF) | Transcriptional regulator |
| comp7262_c0_seq4 | Delay of germination (DOG1) | Transcription regulator |
| comp16233_c0_seq1 | DREB (AP2/ERF) | Transcription regulator |
| comp14632_c0_seq1 | LEA ASRP1 (group 7) | Response to stress |
| comp16047_c0_seq2 | Ubiquitin –associated /TS-N domain | Response to stress |
| comp14615_c0_seq1 | Early light inducible protein 9 (ELIP9) | Response to stress |
| Comp16362_c0_seq1 | Heat Shock protein 70 (HSP70) | Response to stress |
| comp4047_c0_seq1 | Dehydrin (group 2) | Stress-induced hydrophilic protein |
| comp12617_c0_seq3 | Dehydrin (group 2) | Stress-induced hydrophilic protein |
| comp17425_c0_seq1 | Rare cold inducible (RCI) | Stress-induced hydrophobic peptide |
| comp4121_c0_seq1 | Rare cold inducible (RCI) | Stress-induced hydrophobic peptide |
| comp12665_c0_seq1 | LEA 14A(group 5C) | Stress-induced hydrophilic protein |
| comp11969_c1_seq1 | Rubredeoxin-light domain | Unknown |
| comp15430_c0_seq1 | Peroxidase | Oxidative stress |
| comp18775_c0_seq1 | Monothiol glutaredoxin S17 | Oxidative stress |
| comp3950_c0_seq1 | Cycloartenol-c-24-methyltransferase SMT1 | Lipid metabolism |
| comp15145_c0_seq1 | 1,3 betagluconase | Carbohydrate metabolism |
| comp13913_c0_seq1 | Dynein light chain | Cytoeskeleton organization |
